# Is context considered in health inequity research and why? An examination of current trends in Ethiopia

**DOI:** 10.1186/s13010-026-00228-z

**Published:** 2026-10-06

**Authors:** Belayneh Taye Gedifew, Lucinda Cash-Gibson, Endalamaw Tesfa, Behailu Atinafu Dessie

**Affiliations:** 1 Institute of Advanced Study and Department of Philosophy, University of Warwick, Coventry, UK; 2 Department of Political Science and International Studies, Bahir Dar University, Bahir Dar, Ethiopia; 3 Department of Management, Law, Society & Humanities, Pompeu Fabra University-Barcelona School of Management, Barcelona, Spain; 4 Johns Hopkins University - Pompeu Fabra University Public Policy Center (UPF-BSM), Barcelona, Catalonia, Spain; 5 College of Medicine and Health Sciences, Bahir Dar University, Bahir Dar, Ethiopia; 6 Department of Journalism and Communication, Bahir Dar University, Bahir Dar, Ethiopia

**Keywords:** Ethiopia, Healthcare context, Global health, Health inequities, Research methods

## Abstract

**Background:**

Health equity research requires a comprehensive understanding of the historical, social, and political factors that contribute to health disparities. Ethiopia is a nation with a long political and economic history and a diverse culture, encompassing multiple health knowledge systems and traditional healing practices. This background context is essential for research, as the country faces significant health inequities among individuals and across community groups. This study aims to examine the consideration of context in health inequities research, with a focus on Ethiopia.

**Method:**

This study used a Systematic Review Literature followed by Critical Interpretive Synthesis (CIS). Using a systematic search, we identified potential texts for inclusion. We searched three electronic databases, PubMed, HINARI, and Google Scholar, for publications up to June 30, 2023. Then, through systematic analysis followed by critical interpretive synthesis and conceptual translation, we developed a synthesizing argument about the trends in health inequities research.

**Findings:**

Our systematic analysis and CIS reveal that a decontextualized approach dominates health inequity research in Ethiopia. This approach heavily relies on quantitative methods and repeatedly emphasizes wealth, education, and geographic location as key variables. Such emphasis obscures the underlying structural and political causes of inequity. Additionally, this decontextualized research is supported by a political economy of knowledge production that mainly depends on international funders and secondary data, which may prioritize measuring equality over pursuing meaningful equity.

**Conclusion:**

The dominant research approach on Ethiopia focuses on quantitative methods and lacks contextual insight. This approach yields evidence that is inadequate for addressing the root causes of health inequality and inequity, as it unintentionally reinforces the very disparities it aims to eliminate by prioritizing technocratic solutions over transformative ones. Therefore, a crucial shift in epistemology toward contextually grounded, mixed-methods research supported by interdisciplinarity and multidisciplinarity is necessary to generate the knowledge required for meaningful and equitable change in Ethiopia and similar LMICs.

**Supplementary Information:**

The online version contains supplementary material available at https://doi.org/10.1186/s13010-026-00228-z.

## Introduction

The pursuit of social justice and health equity involves addressing deeply rooted social inequities influenced by a complex mix of local and global historical, political, and cultural factors. This issue is highly apparent in Ethiopia, where the country’s diverse socio-cultural and ethnic groups, along with its intricate historical socio-political fabric and frequent foreign military occupations, have shaped its healthcare system, amongst other sectors. The development of modern healthcare in Ethiopia is relatively recent, with the establishment of the first hospital in Addis Ababa by the Russian Red Cross in 1909 being regarded as a significant milestone in the history of modern healthcare services. Then, the Italian occupation (1931–1936) led to a considerable expansion of modern medicine and healthcare services. Since Ethiopia’s liberation from Italian occupation in 1941, an institutional framework for modern healthcare services has been established. Following the 1974 revolution and the end of the monarchy, the military regime (1974–1991) formulated a relatively elaborate health policy aimed at establishing community-based primary health care (PHC) [1].

Nonetheless, the military regime’s achievements were limited by resource scarcity and the ongoing civil war. The rise to power of the Ethiopian Peoples’ Revolutionary Democratic Front (EPRDF) in 1991 coincided with the end of the Cold War. Studies show that, combined with domestic measures, healthcare accessibility expanded significantly for three consecutive decades. The success in mobilizing domestic and foreign resources, along with strong cooperation with development partners and donors, plays a significant role in facilitating changes in PHC [2]. PHC was the foundation for healthcare services during the military and EPRDF regimes, aligned with the “Health for All by the Year 2000” goal of the Alma-Ata Declaration of 1978 [3]. While post-liberation governments attempted to establish comprehensive primary healthcare, notably through EPRDF-era reforms aligned with the Alma-Ata Declaration [2, 3], these efforts have been consistently undermined by the ‘depoliticization’ of healthcare [4, 5], which often masks the underlying power dynamics.

Ethiopia has worked to resist economic reforms driven by the recent Structural Adjustment program, influenced by neoliberal global pressures through its unique private-public divisions of capitalist reforms [6] and by adjusting development aid according to its ‘developmental state’ approach amidst these global influences [7]. Additionally, Ethiopian societal culture remains relatively ‘closed,’ and individuals have experienced less impact from capitalist and Western cultural influences [8]. Despite these efforts, Ethiopian healthcare has undergone various reforms, including decentralization, financing, and human resources [9], mainly influenced by international factors. As a result, Ethiopia’s healthcare system still relies heavily on foreign aid and donations, which make up over one-third of total health expenditures, and faces challenges related to aid fragmentation and misalignment with national priorities [10–12]. Furthermore, the domestic political economy, including factors such as ethnic conflicts, war, political instability, resource limitations, and poverty, as well as government commitment, has significantly affected the healthcare system [13, 14], creating substantial barriers to providing equitable healthcare. This context is essential to understanding the development of health inequalities in Ethiopia.

Ethiopia’s rich cultural diversity, encompassing diverse health epistemologies, is another critical factor influencing the healthcare system and its delivery. For many communities, such as the Konso in southern Ethiopia and those in northern Ethiopia, health and illness are viewed holistically, encompassing social, spiritual, and environmental factors. In this view, illness represents a disruption of social harmony and spiritual imbalance [15, 16], rather than a simple biological disturbance caused by pathogens or physical harm. Similar indigenous health knowledge systems are also found among Ethiopian immigrants in Europe and Jewish immigrants from Ethiopian origin in Israel, often leading to tensions with the Western biomedical system [17]. This highlights that the fundamental ideas of health and disease are relational and deeply rooted in culture [18–20], rendering a holistic, decontextualized biomedical approach inadequate for understanding and addressing health inequities in the Ethiopian context.

Healthcare justice, which demands equity in ‘access, allocation, and the capacity to utilize and benefit from healthcare by individuals and the public’ [21], needs to be applied with sensitivity to the unique socio-political, environmental, and historical context. In this regard, low- and middle-income countries (LMICs) face limited access to healthcare due to multiple factors, including resource scarcity, frequent pandemics and epidemics, and natural disasters. When resources are scarce in these countries, fairly allocating limited resources becomes a critical ethical and policy challenge, involving rationing and distributing resources and services by denying some groups to benefit others, based on different trade-off criteria [22, 23]. This process of fair allocation amid scarcity should never be left ungoverned; the ‘capacity for healthcare utilization’ [21] within individuals and communities should guide it. Regarding health equity, this ‘utilization capacity’ is shaped by broad systemic and social determinants of health, including ‘environmental, socio-economic and political determinants of health’ [24–26], and is influenced by national, regional, and global factors.

This study, therefore, aims to examine how socio-cultural, historical, and political contexts are framed within the field of health inequities research in Ethiopia. Specifically, we critically analyse the methodological and epistemic stances and gaps in published scientific articles on health equity and equality, focusing on Ethiopia, and we offer a systematic analysis and an iterative, interpretive synthesis. The study specifically addresses the following key questions:

- What methodological and epistemological position underpins the research, and how does this influence what is considered evidence?
- To what extent does the research design engage with and incorporate relevant historical, political, socio-cultural, and economic contexts?
- What power dynamics are inherent in the research design, and how are issues of positionality and representation managed in knowledge production on health inequity, considering the political economy of knowledge and the global health inequities knowledge production?

### Context and health inequities research

While acknowledging the importance of context in achieving equity, research aimed at addressing it often restricts itself to including it within its methodological framework. This creates a significant paradox in healthcare research in LMIC settings, as the global health research paradigm is heavily shaped by the Global North [27, 28], which has traditionally emphasized positivist and quantitative methods and biomedical models of health. Such a positivist approach and framing can overlook the unique historical, political, and cultural factors that influence health inequities in each context. This results in a fundamental epistemological issue in research, which neglects context, inevitably yielding evidence that misses the underlying causes.

A conceptual-methodological mismatch between equality and equity is also another challenge in research on health equity. This is true because the dominant research designs are inherently suited to measuring equality, which is a ‘distributive’ concept referring to the equal distribution of resources. The results of these ‘equality’ framed designs are frequently presented with the normative aim of achieving equity. However, equity has a connotation of justice and addresses unjust structural disparities. This conflation of concepts hides the fact that documenting an inequality is not the same as diagnosing the inequity that created it [29, 30]. As a result, this research approach risks producing evidence that guides interventions that are technically effective but weak in transforming systems. This is also a key weakness of the positivist tradition identified in social research on health inequalities [31].

Awareness of health inequities in Ethiopia has grown over the past few decades. For example, national initiatives such as the Health Sector Transformation Plan (HSTP) [32] and the National Health Equity Strategic Plan [33], as well as global agendas such as the UN’s Sustainable Development Goals, have strengthened this commitment. Operationally, health equity is officially mandated by the Ethiopian Federal Ministry of Health (MoH) and promoted through programs such as the Health Extension Program (HEP) [34] and Community-Based Health Insurance [33]. Equity is also a key principle in the national healthcare allocation guidelines. Similarly, a recent bibliographic analysis shows that Ethiopia is one of the least health inequity- and inequality-research-producing countries worldwide, yet among the top producers in low-income countries, particularly with an increase in research production over the past 20 years [35]. However, literature critiques highlight gaps in implementation and research, particularly in neglecting structural, systemic, and political determinants of health. For instance, some argue that the HEP, often praised as a success, demonstrates limited engagement with health system governance frameworks and largely overlooks how state repression and restricted freedoms may distort reporting and undermine the equitable functioning of the health system [5]. Others also demonstrated that health inequity in Ethiopia is often viewed as a technical challenge, focusing on measurable metrics and decentralized, federated solutions. This trend overlooks the influence of structural and political factors crucial to achieving health equity [4].

## Methodology

### Methodological approach

This study conducted a Systematic Review of the Literature followed by a Critical Interpretive Synthesis (CIS). The mixed approach ensures both methodological rigor and conceptual depth, as well as analysis related to our study questions. The established PRISMA framework guided the systematic review to ensure transparent reporting and adherence to the process [36–38]. Following best practices, the process began with formulating straightforward research questions, then progressed through comprehensive database searches, strict inclusion and exclusion criteria, and quality appraisal to reduce bias. Data extraction and synthesis combined quantitative meta-analysis methods with qualitative meta-synthesis approaches, allowing the integration of diverse evidence types.

Building on the systematic review foundation, a CIS was undertaken to move beyond aggregation of findings, interrogating contradictions, gaps, and underlying assumptions in the literature, and situating the evidence within broader theoretical and policy contexts [39–44]. Using these approaches in order, we analysed the production of knowledge about health inequities in Ethiopia’s research trends published up to June 2023. CIS is designed to critically interrogate diverse literature and develop new conceptual frameworks and interpretations. While systematic review analysis often yields summative answers, CIS complements it, making the mixed approach suited to the study’s primary aim: to examine the methodological framing and how socio-cultural, historical, and political-economic contexts are considered within existing research trends. In this respect, CIS provides the necessary methodology to critique the current theoretical landscape, to synthesize a fresh conceptual perspective on health inequity research in the Ethiopian context, and to set a new research agenda as the way forward.

### Literature search strategy, selection criteria, and process

To ensure the review is based on a comprehensive and relevant body of literature, we conducted a systematic search to identify potential texts for inclusion. We searched three electronic databases, PubMed, HINARI, and Google Scholar, for publications up to June 30, 2023. The search strategy combined Medical Subject Headings (MeSH) and free-text terms related to the key concepts: (“Health Equity” OR “Health Equality” OR “Health disparity” OR “Health Inequity” OR “Health Inequality” OR “Health Determinants”) AND (“Ethiopia”). The search was unrestricted by publication date or study type to capture the full scope of the research landscape.

Eligibility criteria were established to select texts that offer insights into how health equity and inequality are understood and studied within the Ethiopian context. In this respect, original research articles that explicitly address health equity, health equality, health disparities, and/or the social determinants of health (and health inequalities) in Ethiopia were included. On the other hand, commentaries, review papers, editorials, study protocols, and articles that are not focused on Ethiopia or do not primarily address inequity or inequality as a central theme were excluded.

**Fig. 1:**
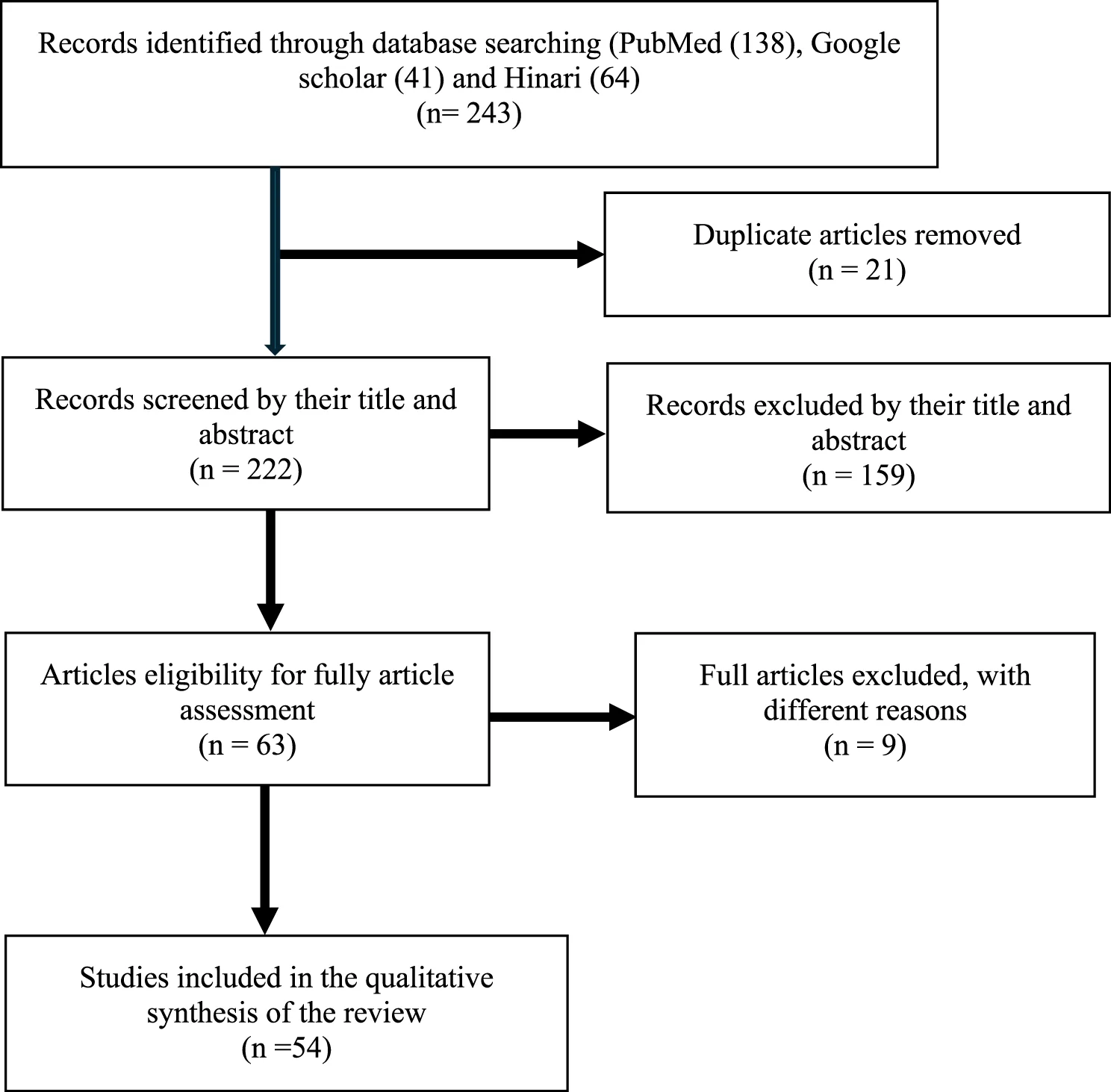
Flow diagram of the study selection process for the critical interpretive synthesis

All records identified through the database search were imported into EndNote X9 citation management software. After removing duplicates, the remaining records’ titles and abstracts were screened for eligibility. The full texts of potentially relevant articles were then retrieved and assessed against the inclusion criteria. This process is detailed in the PRISMA-style flow diagram (Fig. 1).

### Data extraction and management process

Data extraction in systematic reviews involves carefully capturing relevant information from selected studies using structured templates or coding frameworks to ensure consistency and reduce bias [36]. Each research article was coded using a metadata coding sheet and a comprehensive codebook developed for this study before the critical synthesis. Coding was conducted independently by two trained researchers to ensure inter-coder reliability. This process generally includes recording study characteristics, methodological details, outcomes, and contextual variables aligned with the review’s objectives [45]. To support interpretive synthesis, extracted data may also include conceptual themes and theoretical constructs that facilitate deeper analysis across diverse evidence types [38]. Therefore, to achieve a transparent and comprehensive synthesis, key data were extracted, systematically analysed, critically interpreted, and synthesized from each study, then organized into a structured table (see Supplementary Table 4).

The extraction, interpretation, and synthesis incorporate the author’s name and publication year, the authors’ disciplinary backgrounds as indicated by their affiliations, the geographical scope of the study, research design and approach, data collection methods, sample size, key variables analysed, the theoretical framework, the use of conceptual frameworks related to equity and equality, primary findings, and our own epistemic and methodological critical interpretive judgments. Most importantly, we gathered analytical notes on each study’s conceptual, epistemic, and methodological commitments, along with its engagement with contextual factors such as political economy, history, socio-cultural, and environmental influences, which formed the foundation for our critical interpretation.

### The systematic analysis, CIS process, and presentation

Our systematic analysis and CIS were conducted in two stages. Stage one involves descriptive mapping with general descriptive and interpretive implications. This phase includes summarizing the methodological and disciplinary features of all included studies to create a comprehensive overview of the field and research trends. The results of this descriptive analysis and the general interpretive implications are shown in Table 1 in the Results section. This descriptive interpretation and general interpretive implication are further critically interpreted and synthesized in stage two of CIS, which forms the core of the analytical work. In stage two of our CIS, three of the authors engaged in a repetitive process of critically examining and interpreting the texts included in our study. This could ensure the validity of the synthesis process. Critical Interpretive Synthesis involves iterative reading and re-reading of diverse literature to identify recurring themes, contradictions, and conceptual gaps [39]. Rather than simply aggregating findings, CIS interrogates underlying assumptions and theoretical frameworks, allowing for the construction of new synthetic concepts that reflect broader socio-political contexts. This approach is instrumental in complex fields like health equity, enabling researchers to challenge dominant paradigms and generate transformative insights [43].

**Table 1:** Thematic descriptive interpretations and general implications of reviewed studies

Characteristic	Category	Number of Studies	Percentage (%)	General Implication
Methodological Design	Quantitative	44	81.4	Dominance of a positivist paradigm focused on measurement and correlation.
	Qualitative	8	14.8	The qualitative studies are not merely a different “type” of evidence; they are evidence of a different and necessary way of knowing. They demonstrate a consideration contextual inquiry of social and political contexts, which are often overlooked within the broader research trend, and quantitative studies specifically.
	Mixed	2	3.7	Severe lack of methodological triangulation.
Primary Data Source	Ethiopian DHS	25	46.3%	Heavy reliance on a single, large-scale dataset that dictates a national, decontextualized analysis.
	Other Secondary Data	12	22.2%	
	Primary Data Collection	17	31.5%	A tendency to incorporate local voices and disaggregation of data.
Disciplinary Perspective	Health Sciences Only	40	74.1%	Constricted epistemological view, sidelining critical social science perspectives.
	Interdisciplinary	9	16.7%	
	Social Sciences Only	4	7.4%	
	Not Specified	1	1.9%	
Geographic Scope	National	36	66.7%	Tendency towards broad generalizations, missing local and regional contexts and associated factors.
	Sub-National (Regional)	18	33.3%	A tendency to consider regional and local context.
Primary Explanatory Focus	Wealth/Income	38	70.4%	Circular and repetitive analysis focused on individual-level determinants, neglecting systemic drivers and the chains and causal pathways leading to inequities.
	Education	23	42.6%	Circular and repetitive analysis.
	Urban/Rural Residence	22	40.7%	Circular and repetitive analysis.

Following the two stages, we analysed each study using guiding questions that focused on the epistemic and methodological framing of engagement with broader socio-cultural and political-economy dynamics and contexts. We explore how specific socio-cultural and political-economic dynamics, as well as the contextual environment, are incorporated into the methodological framework. We also examine how issues of positionality and representation are addressed in the production of knowledge about health inequities, as well as the power dynamics involved in this framing and the broader research trends. We compared studies, looking for connections, contradictions, and echoes to build a coherent critical argument. Through this process, we developed the synthesizing argument based on the patterns identified across the included studies, that is, ‘a decontextualizing research paradigm characterizes the field and the research trend in Ethiopia.’ The results of stage two CIS are illustrated in Table 2 of the Results section. Acknowledging the flexibility inherent to CIS, we adhered to key reporting practices to enhance the trustworthiness and rigor of the review process [40].

**Table 2:** Conceptual translation and development of the synthesizing argument

Theme No.	Theme Constructs	Illustrative Supporting Studies	Conceptual Translation & Interpretation Process
Theme 1	Hegemony of the Quantitative, Decontextualized Lens	[51, 53, 56, 83]	The statement that “wealth is a key determinant” appeared in these and many other quantitative studies. We saw this a prevailing approach, a decontextualized approach. The repeated emphasis on this correlation, without examining the political and economic factors driving wealth inequality, highlights a core epistemological limitation of the dominant approach.
Theme 2	Disciplinary Narrowness and Constriction of the Research Agenda	[48, 86]	These studies were recognized for claiming an “interdisciplinary” approach, combining health and social sciences. However, we saw their continued reliance on purely quantitative methods as indicative of superficial interdisciplinarity. Instead of adopting a critical epistemological stance, the social science perspective was reduced to variable selection, limiting the research to technical questions.
	Counterpoint to the prevailing Trend	[94]	This qualitative study was seen as a conceptual challenge to the prevailing trend. The clear emphasis on donor influence and political negotiation highlighted the critical, structural questions that the mainstream quantitative health science approach often omits.
Theme 3	The Enduring Triad as Explanatory Default	[52, 57, 88]	In these and many other studies, variables like wealth, education, and residence were viewed not as key social determinants. We also see a sign of methodological individualism, where they focus on individual traits. As a result, they can be taken as examples that overlook failing to identify the root cause, systemic injustice.
Theme 4	The Methodological Short-Circuit and the conceptual conflation and dilemma of Equality and Equity	Most quantitative studies [51, 52, 93]	We saw the focus of these studies on measuring distribution and their shared conclusion of identifying unequal outcomes. While the approach aimed to study *equality*, they consistently framed their study around the normative goal of *equity*.
	Counterpoint to the dominant decontextualized approach	[90, 91]	This study served as an example of contextualizing the triad. It examined a common variable, such as “gender,” not only as a comparison category but also as a structural mechanism of inequality. This offered a counterpoint that allowed us to improve our critique.
Theme 5:	The Political Economy of Knowledge Production: funders and data sources shaping the research frame	[48, 49, 54, 62, 95, 97]	These exemplars show how funders and dependency on a single secondary dataset may create a research cycle that is often decontextualized, oriented toward international priorities, and limited in its ability to generate transformative, locally owned solutions.

### Findings of the systematic review analysis and CIS

In the review, 54 studies were included for an in-depth systematic analysis, critical, and interpretive synthesis. The list of these studies, along with their methodological characteristics, is summarized in Table 3.

**Table 3:** List of included studies (*n* = 54) based on methodological characteristics

Overall Mythological Design	Number and Studies
Quantitative	44 [46–89]
Qualitative	8 [4, 90–96]
Mixed-Methods	2 [97, 98]

We conducted a systematic analysis and CIS to develop a conceptual argument about the epistemic and methodological framing of the consideration of specific contextual factors within the health inequities research trend. In this section, we first present a descriptive overview of the methodological and disciplinary characteristics of the 54 included studies, highlighting the general implications that establish the descriptive-interpretive basis for our synthesis. We then present the core output of the critical synthesis, a synthesizing argument, developed through conceptual translation and illustrated in a conceptual table, that critiques the dominant research paradigm, functional for our later discussion and synthesis.

### Methodological and disciplinary field landscape, and the general interpretive implication

The included studies reveal a field characterized by marked regularity in methodological framing, theoretical and conceptual approaches, and the disciplinary backgrounds of authors. As summarized in Table 3, the research landscape is overwhelmingly dominated by quantitative studies, which constitute 81.4% of the literature. A significant portion of these (46.3%) rely exclusively on secondary analysis of the Ethiopian Demographic and Health Surveys (EDHS). The disciplinary perspective is similarly constricted, with 74.1% of studies situated solely within the health sciences. This configuration, a field defined by quantitative, health science-driven analysis of national survey data, establishes the foundational context for our critical interpretation. Below are methodological and disciplinary features from the authors’ affiliations, as well as the overall implications of health equity studies focused on Ethiopia in Table 3 (*n* = 54).

### The CIS of the decontextualized research approach

Moving beyond the earlier descriptive interpretation of the studies, our CIS demonstrates that these characteristics establish a clear, dominant paradigm with specific epistemological effects. Therefore, based on our iterative process of reading, interpreting, and translating concepts across the studies (see Suppl. Table 4), we argue that the field is limited by a decontextualizing research paradigm that consistently masks the structural, socio-cultural, historical, and political-economic causes of health inequities in Ethiopia, as well as their broader regional and global contexts. This paradigm is reflected in five interconnected themes that helped us develop our core argument- a decontextualizing research approach dominates the field, systematically obscuring the structural drivers of health inequity. The process of creating this argument and themes is shown in Table 2 below.

#### The dominance of the quantitative, decontextualized perspective

Our synthesis the research trend shows a consistent methodological pattern across the field. According to Tables 1 and 81.4% (44/54) of studies used quantitative methods, with nearly half (46.3%, 25/54) relying exclusively on secondary analysis of the EDHS. As a result, research using this data often lacks historical and political context, instead offering a snapshot of differences in wealth, education, geography, and other variables, using a single determinant factor or a combination of 2–3 determinant factors. For example, most studies (*N* = 38) measured the effects of the wealth index on health service utilization, including antenatal care, delivery, postnatal care, reproductive health services, family planning, vaccination, early initiation of breastfeeding, and utilization of newborn and child health services. In these studies, the household wealth index is significantly associated with various health service utilization in the Ethiopian context. Additionally, 22 studies examined geographical inequalities in health, highlighting that place of residence is a key social factor influencing the use of health services, including antenatal care, delivery, postnatal care, reproductive health services, family planning, vaccination, early breastfeeding initiation, and newborn and child health services (see Suppl. Table 4).

This reliance on EDHS, a single large dataset, reflects an epistemological approach focused on identifying statistical relationships between variables such as wealth, education, and residence, and health outcomes at a national level. While the quantitative approach detects inequalities, it is limited in explaining why they continue to exist, an essential question of health equity, a question of justice. Within this frame, equity is operationalized as the measurable distribution of health services or outcomes across predefined population categories. Statistically significant associations constitute evidence. And the goal of analysis is often to identify and quantify disparities, which represent the dominance of a decontextualized research paradigm. Indeed, within this decontextualized research trend, complex social phenomena are frequently reduced to binary variables. For example, ‘media exposure’ is quantified as a simple yes/no measure (e.g. [52]). This approach neglects critical aspects such as content, credibility, and the political economy of information.

The analysis in the reviewed studies primarily concentrates on individual or household-level characteristics (e.g., wealth, education). This approach systematically disregards broader structural, historical, and political systems that contribute to and sustain these disparities. The research frequently presumes that its indicators, such as wealth quintiles and service utilization, are objective and accurate representations of reality. Yet, the construction of these categories or their potential to obscure lived experiences and systemic power relations are not often critically examined (e.g. [51, 75]). Some studies (e.g. [70, 77]), employ a technical systems approach, analysing essential drug availability or program utilization through a lens of measurable inputs and outputs. Still, these studies overlook the critical questions about the political rationing and allocation decisions that create those distributions in the first place.

#### Disciplinary background, theoretical framework, and the research agenda

The interpretation of the disciplinary backgrounds and theoretical frameworks across the included studies reveals the overwhelming dominance of authors from the health sciences, including public health, epidemiology, and medicine. This disciplinary imbalance limits the scope of research questions and results in shallow or absent engagement with social theory. This may thereby diminish the research’s explanatory power and transformative potential. This dominance of health sciences privileges a positivist, biomedical worldview that often frames equity as a technical problem of service distribution. As our analysis shows, there is a clear correlation between authors’ disciplinary backgrounds and a focus on measurable equality (see Suppl. Table 4). Even when studies claim an interdisciplinary background (e.g. [48, 86], list ‘health sciences and social sciences’), our synthesis shows that the research approach is framed from a positivist health science perspective. These studies combine fields but remain trapped within a distributive, quantitative paradigm. As a result, most studies fail to incorporate the critical, structural analyses that disciplines such as political economy, sociology, anthropology, or philosophical ethics would offer. Similarly, studies from a social science background (e.g. [89], still apply the social science perspective within a rigid, individual-focused quantitative paradigm. This demonstrates a constrained application of their disciplinary potential, even in social science research (see Suppl. Table 4).

The theoretical frameworks used are mainly functional and individual-behavioural models, descriptive intermediary (rather than structural) social determinants of health (SDH), and technical and economic frameworks. Frameworks like Andersen’s Behavioural Model (e.g. [60, 87]), and other ‘health services utilization’ theories (e.g. [52, 57]), focus on individual traits and choices, often ignoring the social structures that influence those choices. Many studies (such as [49, 51]) frame their work within SDH, yet in a reductionist way. They treat determinants such as wealth and education as separate, measurable variables rather than as outcomes of deeper political and economic systems. Studies based on ‘health economics’ (e.g. [48, 76]), ‘health systems’ (e.g. [77, 81]), or ‘health statistics’ (e.g. [61]), also tend to adopt a ‘technocratic’ efficiency perspective. These studies document disparities in spending, resources, or outcomes. Still, as noted earlier, they often overlook a political economy analysis of the power dynamics and policy choices that create these disparities.

Nonetheless, there are a few studies that break this pattern by being explicitly framed in ‘Political Economy’[94] and ‘Gender Analysis Framework’ [90]. They show how engagement with critical social theory can illuminate the power dynamics and structural mechanisms, such as ‘donation’ and ‘gender,’ that produce health inequities. These examples show that merging key theoretical perspectives to reformulate research questions can overcome disciplinary narrowness, rather than simply listing multiple fields.

#### Wealth, education, and residence as repetitive explanatory categories

Within the prevailing quantitative approach, there is a recurrent, limited explanatory framework. We call it ‘The Enduring Triad of wealth, education, and geographic location’. Its descriptive, cyclical nature characterizes this model, as it consistently identifies disparities without explaining their underlying causes. As shown in Table 1, most studies focus on three main explanatory factors: Wealth/Income (38 studies, 70.4%); Maternal Education (23 studies, 42.6%); and Urban/Rural Residence (22 studies, 40.7%), analyzed separately or in combination. For instance, many studies (including [62, 66, 72]) consistently identify wealth as the primary driver of inequalities in maternal, child health, and longevity. However, this repetitive correlation occurs without analysing the structural economic policies and power dynamics that create and sustain wealth disparities.

A similar explanatory approach is also applied to health services issues with slight variation, such as measles immunization [53], antenatal care [65, 88], and outpatient visits [76]. Area-based inequalities or regional disparities (e.g. [67, 68, 70, 80], without deeper investigating the historical and political marginalization that produced them. Even studies that combine health and social science perspectives (such as [48, 86]) remain within this cyclical approach of measuring distributive equality. Thus, these studies yield a substantial body of evidence that delineates the symptoms of inequality in greater detail; however, they remain silent about the underlying systemic and structural causes of inequity.

Furthermore, the emphasis on individual attributes in the research trend known as methodological individualism risks portraying health disparities as issues caused by personal shortcomings rather than as outcomes of systemic failure and historical injustice rooted in community and society. As a result, the solutions proposed by such research often focus on ‘correcting’ individuals through measures such as microfinance, literacy programs, and media access, and give less attention to reforming the systems that marginalize them both as individuals and within groups or communities. For instance, when examining regional health inequity and inequality research in Ethiopia’s federal structure and decentralized health system, we observe that regions such as Tigray, Amhara, Oromia, and Addis Ababa receive the most research attention. Meanwhile, Regions such as Somali and Gambella are overlooked, highlighting an imbalance in research focus (see the geographical scope in Suppl. Table 4).

#### The implicit primacy of equality over equity in research framing

An analysis of the ‘equity and equality concept use’ within the theoretical and methodological framing reveals a consistent preference for equality over equity. Although this preference may not always be explicit in the language, it is deeply embedded in research approaches. With their approach, they produce evidence that describes disparities but does not sufficiently address the structural fairness and justice necessary to resolve them. Many studies (e.g. [51, 52, 93]), tend to use ‘equity’ and ‘equality’ interchangeably. This conflation is methodologically essential, as treating these concepts as the same implicitly favours an equality framework that emphasizes sameness in distribution or outcomes. This approach avoids the more complex issue of equity, which involves evaluating whether the distribution is just and fair while accounting for systemic and historical factors. For example, observing ‘no difference’ in contraceptive use between groups (e.g [52]), reflects an equality-oriented perspective. This approach might conceal underlying disparities in access, choice, or quality of care among different population groups.

Many studies explicitly focus on ‘equality measurement’[49, 55, 57] or an ‘equality focus’ [47, 50]. This focus is realized through methods that quantify and compare service utilization, coverage, or outcomes among groups defined by wealth, education, or location. Consequently, the research aims to identify and measure disparities (inequality) rather than to explore the systemic barriers (inequity) that cause them. For example, studies measuring ‘wealth-related inequality’[72, 76] illustrate this approach. They document inequality gradients but do not often analyze the moral or systemic reasons behind them or how to address them through equitable actions.

Even among studies using ‘equity’ language, the underlying methodology often reveals an implicit focus on equality. Studies (e.g. [65, 76], in the equity use category can be criticized for a ‘positivist, indicator-driven approach’ that often treats ‘equity’ as a statistical measure rather than as a matter of justice and structural problems. This suggests that the term ‘equity’ is frequently used as a veneer for a research approach that primarily aims to measure and describe the outcomes of inequality. It pays less attention to analyzing or challenging the unjust systems that produce them. Therefore, one can argue that research framed this way consistently provides evidence of inequality without exploring the underlying mechanisms of inequity.

#### The political economy of knowledge production

An analysis of the ‘Funder’ and ‘Data collection method’ reveals a distinct political economy of knowledge production on health equity, with a focus on Ethiopia. This system is characterized by external donor dependency and a secondary data hegemony. The funding landscape is dominated by international actors, including bilateral and multilateral agencies (e.g., the World Bank [48], UK Aid [97], and the Canadian government [54] private philanthropic foundations (e.g., the Bill & Melinda Gates Foundation, which funds a significant portion of the studies (such as [49, 62], and international NGOs and initiatives (e.g., UNICEF [63], and Amref Health Africa [90].

In the reviewed studies, there is a distinct emphasis on measurable, program-specific outcomes, such as vaccine coverage, contraceptive use, and targeted maternal health services. This emphasis aligns with global health and UN-SDGs, as well as international donor and funder priorities. Moreover, the research and metrics guided by these international agents often prioritize cost-effectiveness and health economics frameworks, as seen in recent WHO and World Bank healthcare allocation metrics. Together, the predominance of external funders in the research trend can create a research cycle that is often decontextualized, oriented toward international priorities, and limited in its ability to generate transformative, locally owned solutions.

Additionally, the data collection method is mainly quantitative, secondary, and largely based on large-scale national surveys such as the EDHS and related datasets. This creates a ‘secondary data hegemony’ with several effects, including repetitive, derivative analysis and evidence that lacks context. It results in a repetitive, circular analysis of the ‘Enduring Triad’ (wealth, education, location), as shown in many studies. Furthermore, heavy reliance on these data might lessen the motivation to develop a strong local research capacity for designing and conducting primary, mixed-methods, or field-based qualitative research. As our critical interpretive synthesis of most studies indicates, secondary data often removes local context, lived experience, and a full understanding of inequalities (see Suppl. Table 4).

Furthermore, the research we reviewed often depoliticizes health inequity by reducing it to a technical issue of service coverage and access gaps. This has been noted in one study [4] included in the review, which claims that the metrication of health equity may detract from the fairness, justice, and morality underpinnings of the concept. Indeed, most of the reviewed studies focus on individual factors, such as wealth and education, and unintentionally view inequity as a matter of personal shortcomings rather than an intricate problem with societal and systemic roots. This individuation of health inequity shifts the focus away from government accountability, corporate influence, and colonial, neocolonial, and other systemic factors at the local, regional, and global levels. A study indicates that the depoliticization of healthcare and research in Ethiopia poses serious risks, aligning dangerously with the ‘success stories’ often promoted by governments and international donors, based on government-generated data. This trend in research legitimates existing power structures rather than critically analysing the system and drawing genuine solutions [5].

## Discussion: The imperative for methodological framing and research agenda reform

Our CIS indicates that research on health inequities focusing on Ethiopia is limited by a prevailing approach that struggles to capture the complex, systemic, and context-specific nature of these issues. Currently, research trends are primarily based on quantitative methods and disciplinary narrowness. The trends also show a persistent focus on individual factors, which often produce evidence that lacks conceptual clarity and context. These gaps, coupled with the political economy of knowledge production, as seen in the overriding influence of international funding agencies, could challenge research progress and its aim of addressing inequities. Mainly, the research could reinforce the inequalities it seeks to address by relying on technocratic solutions that emphasize measuring individual differences while overlooking structural causes [4]. To this end, a reform in the understanding and practice of health inequities research is vital to overcoming these challenges. In this respect, conceptual clarity, a contextually grounded epistemology, and the incorporation of diverse methodological approaches with interdisciplinarity are crucial steps toward advancing research on health inequities in Ethiopia and similar LMICs.

### Conceptual advances and epistemic justice

Health equity serves as both a moral principle and an empirical challenge. Thus, it necessitates a clear distinction between inequality, the distribution of health outcomes, and inequity, which refers to unfair health disparities rooted in ethical concepts of fairness and opportunity [99]. It involves preventing unjust and avoidable health disparities, often driven by social, economic, and cultural disparities [100]. Indeed, recent advances in health equity emphasize transitioning from merely describing disparities to investigating the foundational social structures that produce them. A significant distinction exists between health inequalities, which refer to differences in health outcomes, and health inequities, which are avoidable and unjust disparities. These inequities are socially constructed through factors such as socio-cultural, environmental, racial, political, and economic stratifications [30, 101]. Clarifying the distinction between inequality and inequity, along with considering life-course and contextual influences, improves both methodological accuracy and ethical comprehension [102].

More than a simple equitable distribution of health outcomes, health equity is now regarded as a transformative process aimed at redistributing power, resources, and knowledge [103]. Thus, the way we measure disparities, using absolute versus relative indices, reference groups, and analysis levels, shapes how inequities are perceived and addressed [104]. Despite this, conceptual clarity remains limited, as many studies misuse ‘inequality’ and ‘inequity’ interchangeably [105, 106]. Indeed, our analysis of Ethiopia also shows this methodological dilemma. The literature often prefers the concept of equality over equity, frequently confusing the two in both language and methods. This conceptual ambiguity in the dominant research trend can perpetuate epistemic injustice, overlooking the deeper ethical and political issues of fairness. Therefore, for Ethiopia, clarifying the difference between equity and equality is both a conceptual task and a methodological and political necessity. Without this clarity, evidence might remain merely descriptive, limiting the research’s ability to guide policies and interventions.

### Contextually grounded epistemology and method

Contextual factors play a decisive role in producing inequities. Evidence shows disparities are shaped not only by individual behaviours but also by community poverty, social capital, patient-physician interactions, and historical injustices, such as colonial policies and residential areas [101, 107–109]. Global forces, including trade agreements, labour standards, and poverty reduction initiatives, further embed inequities within broader structures of power and governance [107]. Studies also show that health equity cannot be separated from the distribution of power, money, and resources, nor from the political processes by which health is framed and becomes an agenda. For example, comparative studies indicate that social democratic welfare states, higher public spending, fair trade, compulsory education, and microfinance improve health, while neoliberal restructuring and austerity exacerbate inequalities [110, 111]. In addition, studies reveal that social democratic regimes redistribute resources more effectively and achieve lower infant mortality, whereas structural adjustment programs and privatization constrain public health capacity [112, 113].

Furthermore, contemporary challenges such as climate change, digital divides, and colonial legacies demonstrate that health equity is not static but continually reshaped by power, governance, and global economic systems [114, 115]. Case studies from South Africa and England show that entrenched neoliberal reforms widened inequities despite commitments to universal coverage [116, 117]. Ultimately, disparities are driven by structural factors, such as power relations and social policies, that shape income distribution and health outcomes. Research-wise, therefore, the context where health inequity is created needs to be explicitly theorized and framed within the methodology. Specifically, equity needs to be linked to life-course perspectives, historical legacies, and cultural factors, such as norms of solidarity and collectivism. These elements shape how societies distribute resources [107, 118].

In Ethiopia, reliance on quantitative methods using secondary data, narrow disciplinary focus, and attention to individual-level factors produce decontextualized evidence. This may be due to the research trend’s dependence on external funding. Studies show that donor financial leverage and technical expertise affect the power to set research agendas [119, 120], often overriding government priorities in low-income countries [121]. Private philanthropies, notably the Gates Foundation, influence global health agendas through data infrastructures and advocacy platforms [119]. More broadly, funding flows remain concentrated in high-income countries, perpetuating epistemic injustice and marginalizing LMIC researchers [122]. Philosophical analyses link these dynamics to struggles for epistemic freedom and decolonization [123]. Recent calls emphasize embedding epistemic justice in grant-making through participatory agenda-setting and equitable budgets [124], while multi-country evidence shows donor practices often distort national research agendas [120].

Such reliance on external research funding and weak health system capacity in Ethiopia could influence methodological approaches, inclining research towards a quantitative, decontextualized approach. Such an approach in research can reinforce inequities by supporting ineffective, technocratic solutions. To address these issues, research should integrate local lived experiences and cultural worldviews, as well as regional, national, and global political-economic forces, into the framing. Certainly, framing the research agenda in this way requires building national research capacity and establishing an autonomous agency to set the research direction locally.

### Methodological pluralism and mixed methodological approach

Globally, methodologies for addressing health inequality and inequity have evolved from simple surveys to advanced tools. The advanced tools include concentration indices, decomposition techniques, and fixed-effects models that address contextual complexities in community health research [125, 126]. Traditional epidemiological measures, such as absolute versus relative indices, reference points, and concentration curves, remain essential but are insufficient if disconnected from political realities [127, 128]. In this regard, researchers advocate for multilevel, systems, and transdisciplinary approaches to disentangle complex causal chains, integrate qualitative and quantitative data, and address complexities and loops in research [129]. Still, the current guidance for evidence synthesis on health inequalities lacks consensus and does not effectively model how social traits interact to produce inequities. As a result, significant methodological gaps remain [105].

Nonetheless, within the earlier methodological dilemma and pluralism, employing a mixed-methods approach enhances quantitative and qualitative, decontextualized analysis by incorporating the lived experiences [130]. In this regard, for Ethiopia, using a mixed-methods approach is indispensable given systemic, socio-cultural, and environmental factors that could have created health inequalities. For example, in a parallel mixed-methods approach, quantitative data reveal disparities, while qualitative methods, such as a participatory approach, can help explore underlying causes, cultural perspectives, and power relationships. As evidence shows, collaboratively developed research with communities ensures relevance and contextual understanding. This methodological diversity promotes triangulation, interdisciplinarity, and reflexivity, situating equity research within socio-cultural, historical, and systemic frameworks.

### Toward an interdisciplinary roadmap

A shift towards contextually grounded research, a mixed-methods approach, and a reform of research framing is needed. In this respect, to advance grounded reform, we propose a strategic framework grounded in three main pillars.

First, at the national system level and within academic and research institutions, it is crucial to encourage meaningful interdisciplinary collaboration and build capacity to conduct this type of research. This involves moving beyond superficial inclusion of the social sciences and humanities in health sciences to construct deep, equitable partnerships with fields such as political science, geography, anthropology, philosophy, history, psychology, language, and communication and cultural studies. These disciplines provide the theoretical tools needed to examine issues of power, critique neoliberalism, interpret historical contexts, analyze diversity, cultural influences, language and communication barriers, and the cultural foundations of medicine, health, and healing, elements that are notably missing in the current literature.

Secondly, direct research efforts toward understanding the historical and systemic roots of inequities in Ethiopia, including healthcare allocation and injustice. As evident from the reviewed studies, some of the main gaps are the lack of research on the principles and processes of healthcare resource allocation and rationing, as well as insufficient deep analysis of healthcare inequities within the political economy and across national, regional, and global levels. Future research should explore the bureaucratic, political, and ethical factors that influence how resources are distributed among groups, individuals, communities, nations, and regions in Africa and worldwide, including their connections to Ethiopia. This requires moving beyond mere outcome measurements and adopting other methodologies to undertake a more comprehensive exploration of these issues, considering the context they appear within the research’s epistemic and methodological framing.

Thirdly, build health systems research capacity and local autonomous agency in research agenda framing, and parallel to this, support and promote incentives for contextual, mixed-methods research. Funding agencies, academic institutions, and scholarly journals should actively support and incentivize this type of research. This includes giving equal importance to detailed, contextual case studies and large-scale studies, encouraging long-term ethnographic research, and making space for investigations that challenge dominant interests and produce complex, non-generalizable results.

## Limitations

This study has some limitations. First, while the PubMed, HINARI, and Google Scholar databases cover a wide range of health and interdisciplinary literature, relevant studies in other databases might be overlooked. Second, our CIS is naturally influenced by our critical judgment, theoretical perspectives, and epistemological stance. Although we aimed to remain rigorous and transparent throughout our process, other researchers with different theoretical approaches might interpret our results differently. Third, this synthesis, which focuses only on published, peer-reviewed empirical literature, might unintentionally exclude valuable insights from grey literature, such as doctoral theses, institutional and government policies, and evaluations. Finally, although our findings are likely applicable to other LMIC contexts, the synthesis is explicitly based on the Ethiopian context.

## Conclusion

Our findings show that a decontextualizing approach dominates health inequity research in Ethiopia. This approach depends heavily on quantitative methods and repeatedly emphasizes wealth, education, and geographic location. Such emphasis overlooks the deeper structural and political causes of inequity. Moreover, this narrow research focus is supported by a political economy of knowledge production that relies on international funders and secondary data, which may prioritize measuring equality over pursuing meaningful equity. This dominant approach generates evidence that may be inadequate to address the root causes of health inequality and, in doing so, unintentionally reinforces the disparities it aims to eliminate by favouring technocratic solutions over transformative ones.

From a research and policy perspective, achieving health equity in Ethiopia requires more than just technical solutions or improved measurement of disparities. It calls for a fundamental shift toward embracing methodological diversity, epistemic justice, and a critical examination of the political and economic systems that create and maintain inequality. In fact, by adopting a contextually grounded, mixed-methods, and interdisciplinary approach, researchers can help build a stronger, more relevant, and transformative body of evidence that promotes meaningful actions toward a more equitable health future for all.

Moving forward, it is therefore crucial to go beyond complacency with isolated disciplinary approaches and adopt a transformative research agenda that includes interdisciplinary design, methodological diversity, awareness of historical and political economy contexts, and a strong commitment to social justice. This guarantees that research and research practices do not perpetuate epistemic injustice by silencing local knowledge, community experiences, and voices. Consequently, research institutions, stakeholders, government agencies, and funders should promote a comprehensive, critically aware, and contextually sensitive approach to research addressing health inequities. Through this, researchers can generate compelling evidence to understand the structural causes of inequality, inform policies and interventions, and build more just societies.

## Electronic Supplementary Material

Below is the link to the electronic supplementary material.

Supplementary material 1

## Data Availability

The authors declare they can provide data related to this study upon request.
