# Supplementary material for: Is context considered in health inequity research and why? An examination of current trends in Ethiopia
Source: Philos Ethics Humanit Med. 2026 Oct 6;21:29. doi: 10.1186/s13010-026-00228-z (PMC13644288; doi:10.1186/s13010-026-00228-z)
Supplement: Supplementary file 1 [file 13010_2026_228_MOESM1_ESM.docx]

**Supplementary Table 4:** Systematic Analysis and Critical Interpretive Synthesis of Health Equity Research in Ethiopia - Comprehensive Analysis

| **No.** | **Authors** | **Study area** | **Funders** | **Data collection method**  **(primary or secondary data)** | **Sample size** | **Disciplinary background** | **Theoretical Framework** | **Equity/Equality Concept Use** | **Key Variables Analysed** | **Main Findings** | **Epistemological/Methodological Critique** | **Synthesis Theme** |
| --- | --- | --- | --- | --- | --- | --- | --- | --- | --- | --- | --- | --- |
| 1 | Accorsi et al. (2017) | Oromia | “Italian Contribution to the Health Sector  Development Programme and Contribution to the MDGs  Fund | Quantitative (Secondary) | 105,025 | Disciplinary background not clear | Not specified | equality focus | Gender Vs indication for admission, cause of death, and Fatality rate | Gender inequalities do exist in rural, remote settings, but tend to affect women differently | Quantitative Hegemony: It adheres to positivist epistemology. It lacks engagement with gender as a social construct, shaped by power, norms, and institutional structures. | Hegemony of Quantitative Lens  (Canonical Tripartite explanatory) |
| 2 | Ahmed & Seid (2020) | Nation  Wide | Not Mentioned | Quantitative (Secondary) | 67876401 women (4061 from rural and 2340 from  the urban area. | Health sciences | Health services utilization | Terms used interchangeably | family planning  messages on radio, television, or print media (eg, magazines  and newspapers), | No difference in contraceptive utilization between media-exposed and non-exposed women in urban and rural areas | Decontextualized Variables: Treats "media exposure" as a binary variable, ignoring content, credibility, and the political economy of information | Hegemony of Quantitative Lens, Enduring Triad |
| 3 | Amare et al. (2022) | Nation-wide | Not Mentioned | Quantitative (Secondary) | 15,106 | Medicine and health sciences | Social determinants of health | Terms used interchangeably | Wealth, education, residence, subnational status, and breastfeeding initiation | In Ethiopia, the wealth-, residence-, and educational-related inequalities of EIBF increased  significantly between the years 2000 and 2011 but disappeared in the 2016 survey year. | Methodological Individualism: Grounded in a positivist epistemology, it privileges quantifiable indicators. This constrains understanding of cultural, relational, and normative dimensions of breastfeeding practices. | The Enduring Triad/ (Canonical explanatory model) |
| 4 | Ambel et al. (2017) | Nation wide | The World Bank | Quantitative (Secondary) | 45000 | Health sciences and social sciences | Health economics | Equality focus stated | Wealth, health service utilization | The changes observed in wealth-related inequality tend to  reflect the changing direct effect of household wealth on child health and health service use. | Limited Interdisciplinarity: Combines health and social sciences but remains within a distributive paradigm without political economy analysis | Disciplinary Narrowness |
| 5 | Beaumont et al. (2023) | 4Regions  (Amhara, Oromia, SNNP&Tigray | Bill& Melinda Gates Foundation | Quantitative (Primary) | 2189 | Health sciences | Framed in Social Determinants | Equality measurement | Socio-economic status, postnatal care | Postnatal care in Ethiopia: evidence of socio-economic inequality in  both coverage and quality. | Individual-focused Analysis: Uses primary data but focuses on quantifiable service metrics, omitting experiential and cultural dimensions of postnatal care. | Hegemony of Quantitative Lens, Enduring Triad |
| 6 | Bergen et al. (2018) | Jimama  Districts | Jimma University, University of Ottawa+Child Health in Africa initiative+Canada’s International Development Research Centre | Qualitative (Focus group and interviews) | 12FGD(612participants | multidisciplinary center and health sciences | Framed in Social constructionism | Equity/equality is used interchangeably | Perceptions, experiences, and health inequalities | Perceptions and experiences related to health and health inequalities | Contextual Engagement (Exception): Actively explores lived experiences and perceptions, providing crucial insights beyond quantification | Positive Exemplar, but small data set |
| 7 | Bergen et al. (2021) | Nationwide | Not mentioned | Qualitative (Interview + Document Analysis) | 23 KIs & 6 PDR | Health sciences | The WPR approach | Equity language used | Health service coverage, efficiency | Health inequity is a normalized and inevitable concern that is regarded as actionable.  (can be altered) but not fully resolvable (can never be fully achieved). | Technocratic Framing: Frames health service expansion in terms of economic productivity, reflecting a depoliticized understanding. | Positive exemplar showing the contextual underpinnings |
| 8 | Bergen et al. (2019) | Nationwide | Nationwide | Qualitative (Interviews) | 12 | Health sciences | Geographic analysis | Equality measurement | Geographic factors, health service inequalities | Geographic factors are predominant for health service inequalities | Geographic Determinism: Identifies geographic factors without critical analysis of the historical and political decisions that led to unequal distribution. | Positive Exemplar, but limited to descriptive analysis |
| 9 | Bergen et al. (2020) | Nation  Wide | Jimma University, University of Ottawa+Child Health in Africa initiative+Canada’s International Development Research Centre | Qualitative (Interview) | 17 | Multidisciplinary center and health sciences | social determinants of health and health equity | Explicit equity focus | Gender inequality, health service utilization | Efforts to address gender inequalities were synonymous with the promotion of  a women’s health agenda, which was largely oriented towards promoting health service  use. | Structural Analysis (Exception): Explicitly addresses gender inequalities as a structural mechanism rather than an individual variable | Positive Exemplar |
| 10 | Biadgo et al.2021 | Not Funded | Oromia, Tigray, Amhara &SNNP | Quantitative + Qualitative (Mixed) | 1920 | Health sciences | Health Service Utilization | Equality oriented | Input, process, output on maternal and neonatal health care | Quality of maternal and neonatal care gaps were observed in the input, process, and output | Though it was highlighted that the study employed a mixed design, the analysis highly depends on quantitative findings | Hegemony of Quantitative Lens |
| 11 | Bobo et al., 2020 | Not Mentioned | Nation  wide | Quantitative (Secondary) | 1,930 | Health sciences | Social Determinants of Health | Equality Focused | Family planning, antenatal care, socioeconomic status, exposure to media, geography | maternal  health services such as family planning and antenatal  care, socioeconomic status, exposure to media, urban  Rural residence and maternal education explain inequalities  in basic vaccination coverage in Ethiopia. | Descriptive analysis of socio-economic factors overlooking cultural practices | Hegemony of Quantitative Lens |
| 12 | Borde et al., 2020 | NORHED | Southern Ethiopia | Quantitative (Primary) | 2350 | Health sciences | Economic Determinism/Factor | Equality Framed | Socio-economic parameters | health inequity in the household’s budget share of total OOP  Healthcare payments in southern Ethiopia were high. | Economic Analysis: Focuses on financial aspects without the broader political economy of healthcare financing. | Hegemony of Quantitative Lens |
| 13 | Carasso et al. (2009) | Amhara | Not Mentioned | Quantitative (Primary) | 5 Health centres | Health sciences | Health systems | Implicit equality focus | Essential drugs availability | Availability of essential drugs in budgetary health facilities | Technical Systems Approach: Examines drug availability without analysis of allocation policies or rationing. | Hegemony of Quantitative Lens |
| 14 | Daka et al. (2020) | Oromia | Not mentioned | Quantitative (Secondary) | 5,701 | Health sciences and social sciences | Social determinants | Equity language used | Residence, education, wealth, maternal services | Urban, educated, and rich individuals are more likely to utilize maternal services like ANC, Facility delivery, and postnatal care. | Limited to socio-economic analysis. Combines perspectives but remains within a distributive, quantitative paradigm. | Disciplinary and methodological Narrowness |
| 15 | Debie et al. (2020) | Nationwide | No fund | Quantitative (Secondary) | 3,791 | Health Systems and Policy Department/health | Health policy | Equity language used | Wealth, place of delivery, antenatal care coverage, residence, media | Wealth, place of delivery, antenatal care coverage, residence, maternal education, and media exposure are determinants of children's vaccination inequalities | Policy-oriented but Decontextualized: Policy focus without engagement with the political context of implementation. | The Enduring Triad |
| 16 | Degefa & Hoelscher (2020) | Nation  Wide | Not Mentioned | Qualitative (Interviews) | 27 | Social science (development studies and peace studies) | Framed in Political Economy | Equity focused | Donors' role, universal health coverage | Donors' role in achieving equitable and universal health coverage in Ethiopia | Political Economy Analysis (Exception): Explicitly engages with power dynamics and global influences on the health system | Positive Exemplar |
| 17 | Dessalegn et al. (2020) | Afar | Amref Health Africa in Ethiopia. | Qualitative (Focus group and interviews) | 8 FGD & 16 KIs | Gender Analysis Framework | Gender studies | Equity focus stated | Women's decision-making, resources, and early marriage | Women have poor decision-making, are unable to control resources, are forced into early marriage, and have poor service utilization | Gender Analysis (Contextual): Examines gender power dynamics within a specific cultural context (Afar). | Positive Exemplar |
| 18 | Gebre et al. (2018) | Nationwide | PAN Africa University Life and Earth Science  Institute, University of Ibadan, Ibadan, Nigeria. | Quantitative (Secondary) | 15,525 | Health science (public health and obstetrics and gynaecology) | Public health | Equity language used | Wealth, antenatal care coverage, skilled birth, and postnatal care | The main determinants of inequities were low economic status, illiteracy, rural  residence, no occupation, and less access to mass media. | Wealth Focus: Reiterates wealth disparities without analysis of the structural reasons for these disparities. | The Enduring Triad |
| 19 | Geweniger & Abbas (2020) | Nationwide | Not Mentioned | Quantitative (Secondary) | Not specified | Health science (such as public health, the department of infectious diseases) | Infectious disease | Equity language used | Household wealth, region, maternal education | Children from poorer households, rural regions of Afar and Somalia, who had no maternal education, had lower full vaccination coverage. | Regional Disparities: Documents geographic patterns without historical or political analysis of marginalization. | Hegemony of Quantitative Lens, Enduring Triad |
| 20 | Hailu et al. (2021) | Nationwide | International Development Research Center. | Quantitative (Secondary) | Not specified 23,393 households | Global public health/Centre for ethics and Priority Setting/multidisciplinary / Benefit analysis Incidence analysis | Ethics and priority setting | Equity focus stated | Government spending, health centres | Inequalities in government spending in health centres and hospitals | Resource Allocation Focus: Examines spending disparities, but with limited analysis of the political rationality behind allocation. | Hegemony of Quantitative Lens |
| 21 | Hendrix et al. (2023) | Nationwide | Not Mentioned | Quantitative (Secondary) | Not specified | Global health/ethics/ more multidisciplinary | Global health ethics | Equity language used | Residence, wealth, region | Health access inequalities in rural vs. urban and in poor vs. rich households in different regions | Access Focus: Documents access disparities without a critique of the system that produces them. | Hegemony of Quantitative Lens, Enduring Triad |
| 22 | Jones et al. (2020) | Afar, Amhara & Oromia | UK aid  from the UK government. | Quantitative + Qualitative (Mixed) | 6800 | Social science | Global Evidence:  Gender and Adolescence ( | Equity framework | Gender inequality, disability, education, and water access | Lack of knowledge, information, clean water, and preventive health care services leads to gender inequality and disability. | Methodological Pluralism (Exception): Effectively connects quantitative patterns with qualitative insights about structural barriers. | Positive Exemplar |
| 23 | Karim et al. (2015) | Nationwide | Not Mentioned | Quantitative (Secondary) | 3932 & 3867 | Health sciences | Health services | Equity language used | Education, age, wealth, distance | Education, age, wealth, and distance-related inequities are observed in MNCH. | Standard Determinants: Repeats well-established correlations between individual/household variables and outcomes. | The Enduring Triad |
| 24 | Kifle et al. (2021) | Nationwide | No external Funding | Quantitative (Secondary) | 42,460 | Health sciences | Health economics | Equality measurement | outpatient visits, socioeconomic status | Wealth-related need is the determinant in outpatient care utilization inequality. | Economic Determinism: Focuses on wealth as a primary determinant without the broader political economy of healthcare financing. | The Enduring Triad |
| 25 | Kotiso et al. (2020) | Gombora Districts, SNNP | Not mentioned | Quantitative (Primary) | 796 | Health sciences | Behavioural model | Equality | Food security, education, knowledge, wealth, ANC utilization | Food security, education, knowledge, and wealth are determinants affecting ANC service utilization | Technical Approach: Focuses on individual behavioural determinants without examining systemic food security policies | The Enduring Triad |
| 26 | Kurji et al. (2020) | Oromia | Jimma University, Innovating for Maternal and  Child Health in Africa initiative (co-funded by Global Affairs Canada (GAC),  the Canadian Institutes of Health Research (CIHR) and Canada’s International | Quantitative (Secondary) | 3,758 | Health sciences | Public health | Equity language used | Poverty, education, antenatal care coverage, PNC | Poverty and low education coverage affect antenatal care coverage, PNC, and delivery service utilization. | Poverty Focus: Documents poverty effects without analysis of the political and economic causes of poverty. | The Enduring Triad |
| 27 | Memirie et al. (2016) | Nationwide | Not mentioned | Quantitative (Secondary) | 9,861 & 11,654 | Health sciences | Health services | Equity language used | Wealth, maternal and child health | Socioeconomic factors were the  The main predictors of differences in maternal and child health services utilization and child health outcomes. | Wealth Correlation: Reiterates wealth disparities as a primary finding without deeper investigation. | The Enduring Triad |
| 28 | Mengist et al. (2022) | Nationwide | No specific funding received | Quantitative (Secondary) | 3,952 | Health sciences | Public health | Equity language used | Residence, wealth, education, parity | Rural residence, being poor, less education, and grand multi-para are the determinants for late antenatal care coverage service utilization. | Standard Model Application: Applies the conventional determinant model without theoretical or contextual innovation. | The Enduring Triad |
| 29 | Miller et al. (2014) | Oromia | Not mentioned | Quantitative (Primary) | 3,200 | Health Sciences | Social determinants | Equality measurement | Residence, gender, wealth, religion | Residence, gender, wealth, caretaker age, and religion are determinants of child health coverage inequalities. | Multiple Determinants: Documents multiple individual and household-level factors without integration into a structural analysis. | Hegemony of Quantitative Lens, Enduring Triad |
| 30 | Mirkuzie et al. (2014) | Addis Ababa | Not mentioned | Qualitative (Interviews) | 21 IDIs & 26 FGD | Health Science | Health Systems | Equity language used | Migrant status, skilled birth, PNC | Migrant women faced inequalities in skill birth and PNC services | Vulnerable Groups Focus (Contextual): Identifies disparities for a vulnerable group with qualitative insight into their experiences. | Positive Exemplar (Partial) |
| 31 | Mohammed et al. (2019) | Nationwide | No specific Funding Received | Quantitative (Secondary) | 8,855 | Health Sciences | Public health | Equity language used | Poverty, education, residence, birth size | Being poor, caregivers' education status, residence, and birth size are the determinants of stunting. | Nutrition Focus: Identifies child nutrition disparities without analysis of the broader food system or political economy of nutrition. | The Enduring Triad |
| 32 | Oh et al. (2019) | Nationwide | Not mentioned | Quantitative (Secondary) | 45,180 | Social science | Social science | Equality measurement | Education, financial factors, travel distance | Education, financial factors, and travel distance are determinants of health care utilization. | Social Science Perspective: Applies social science variables but remains within an individual-focused, quantitative paradigm. | Disciplinary Narrowness |
| 33 | Olsen et al. (2021) | Nationwide | MD-Ph.D. research track program at the  University of Bergen. | Quantitative (Secondary) | Not specified | Health sciences | Health Economics | Equity language used | Region, childhood mortality | Childhood mortality inequalities existed in regions | Modelling Approach: Uses technical modeling without engagement with the political or historical causes of regional disparities. | Hegemony of Quantitative Lens |
| 34 | Seboka et al. (2022) | Nationwide | No funding received | Quantitative (Secondary) | 26,702 | Public Health &Geo special | Health Care | Equity language used | Wealth index, media access, number of children | Wealth index, media access, and number of under-five children are determinants of inequalities in maternal care utilization. | Social Science Application: Incorporates social science variables but remains within a quantitative, distributive analysis limited to uncovering spatial factors | Limited to correlating maternal care with geospatial features |
| 35 | Shibre et al. (2020) | Nationwide | No Fund Received | Quantitative (Secondary) | Not specified | Health sciences | Health services | Equity language used | Socio-economic status, area-based inequalities | Socio-economic status and area-based inequalities are determinants of caesarean section service utilization. | Service-specific Analysis: Focuses on a specific clinical service without a critique of the system influencing its provision. | The Enduring Triad |
| 36 | Shibre et al. (2020) | Nationwide | No funding was received for this work | Quantitative (Secondary) | 7,951  16,515 | Health sciences | Public health and Epidemiology | Equity language used | Wealth, education, and residence | Wealth, education status, and place of residence are determinants of measles immunization coverage. | Vaccination Focus: Identifies immunization disparities without analysis of the program's political economy or community engagement. | The Enduring Triad  Hegemony of Quantitative Lens  Positivist approach via quantitative survey data assumes objective measurement of inequalities |
| 37 | Spangle et al. (2013) | Amhara & Oromia | Support for this work was provided by the Bill and Melinda Gates Foundation, the Ethiopian Federal Ministry of Health, the Oromiya and Amhara Regional Health Bureaus, and the MaNHEP implementing team. | Quantitative (Secondary) | 1,019 | Health sciences | Maternal health | Equality measurement | Wealth, maternal and newborn health | Wealth is the determinant of maternal and newborn health program utilization. | Program Evaluation: Focuses on wealth disparities within a specific program context. | The Enduring Triad: Hegemony of Quantitative Lens  Focused on the usual structural inequalities |
| 38 | Tarekegn et al. (2022) | Nationwide | The Addis Continental Institute of Public Health provided support to cover the researchers. | Quantitative (Secondary) | Not specified | Health sciences | Health services | Equity language used implicitly grounded in the health equity framework | Wealth, residence, education | High wealth, urban residence, and attending education foster skill birth service utilization. Persistent disparities indicate need for targeted efforts to reach rural and poorer populations. | Service Utilization: Standard determinants analysis for a specific health service .Adopts a positivist, quantitative paradigm assuming measurable, objective indicators of equity via survey data, | The Enduring Triad: Hegemony of Quantitative Lens |
| 39 | Teferi & Schröders (2023) | Nationwide | Not specific Funding:  Open access publishing funding provided by Umea University | Quantitative (Secondary) | 8,811 | Health sciences | Public health/ Explanatory pathways for health inequalities framework | Equity language used/employs an equity lens to dissect urban-rural inequalities | Residence, wealth, marital status, parity, Cultural-behaviour, Psychosocial | Residence, wealth, marital status, and parity are determinants of the unmet need for family planning inequalities. | Family Planning Focus: Applies standard analytical model to family planning services./Positivist quantitative approach via decomposition assumes inequalities are objectively decomposable into pathways | The Enduring Triad: Hegemony of Quantitative Lens |
| 40 | Tizazu et al. (2022) | Nationwide | This study was funded by UNICEF Ethiopia. | Quantitative (Secondary) | Not specified | Health science and Social science/Nutrition, public health, and epidemiology with a focus on child feeding practices and health equity in low-income settings. | Nutrition/Implicitly grounded in the UNICEF conceptual framework for malnutrition | Equity language used/equity lens to assess inequalities | Socio-economic status, complementary feeding | Complementary feeding and food group inequalities existed among children of different socio-economic statuses/Widening socioeconomic and geographic inequities in dietary diversity during complementary feeding in Ethiopia | Nutritional Inequalities: Documents feeding disparities without analysis of the food system or political economy of nutrition./Positivist, indicator-driven approach | The Enduring Triad: Hegemony of Quantitative Lens |
| 41 | Tranvåg et al. (2013) | Nationwide | Not mentioned  Norwegian Research Council | Quantitative (Secondary) | Not specified | Health Economics | Public health/Social determinants of health | Equality measurement | Wealth, longevity, life expectancy | Wealth is the determinant of longevity and life expectancy | Longevity Focus: Examines the wealth-mortality relationship without historical or political context. | The Enduring Triad: Hegemony of Quantitative Lens |
| 42 | Tsegaye et al. (2022) | Nationwide | Addis Continental Institute of Public Health has funded this research. | Quantitative (Secondary) | Not specified/Weighted pooled sample of 53,160 women | Health sciences | Health services/Implicitly anchored in the WHO Health Equity Framework | Equity language used | Wealth, education, residence, region | Wealth, education, and residence are determinants of antenatal care coverage | antenatal care coverage Focus: Standard determinant analysis applied to antenatal care coverage./Positivist, indicator-driven approach without exploring structural violence or gendered barriers in access. | The Enduring Triad: Hegemony of Quantitative Lens |
| 43 | Wilunda et al. (2013) | Oromia | This study was funded by Doctors with Africa CUAMM. | Quantitative (Secondary) | 760 | Health sciences and statistical sciences | Health statistics | Equality measurement | Wealth, residence, and emergency obstetric care | Wealth and urban residence are determinants of emergency obstetric care utilization. | Emergency Care Focus: Analysis of a health service but limited to standard individual/household variables. | The Enduring Triad: Hegemony of Quantitative Lens |
| 44 | Woldemichael et al. (2019) | Nation  Wide | Not-Mentioned | Quantitative (Secondary) | Panel data from 11 regions | Health sciences | Health systems | Equity is implicitly implied under Equality  Focus | healthcare workforce,  infrastructure, outcomes, and finance | Disparities in the availability and accessibility of health centres and skilled health workers compared to WHO standards | Geographic Analysis without Political Context: Documents geographic disparities without analysing political decisions, creating distribution patterns | Hegemony of Quantitative Lens |
| 45 | Woldemichael et al. (2019) | Nationwide | Not-Mentioned | Quantitative (Secondary) | Panel data from 11 regions | Health sciences | Health systems | Equity language used | Health professionals, institutional distribution | High health care inequalities are observed with respect to health professionals and the distribution of health institutions | Workforce Distribution: Documents human resources disparities without policy or political economy analysis of workforce management. | Hegemony of Quantitative Lens |
| 46 | Wondimu et al. (2020) | Nationwide | A.W. was funded by the Netherlands Organization for International Cooperation in Higher Education (Nuffic) for his PhD study, grant number: NFP—PhD.16/0015. The funder had no role in study design, data collection and analysis, decision to publish, or preparation of the manuscript | Quantitative (Secondary) | 2,004/3,760 | Health sciences and Social sciences | Health services/Andersen's Behavioural Model of Health Services Use | Equity language used | Antenatal care coverage use, institutional delivery, and media exposure | Antenatal care coverage use; institutional delivery, media exposure, and maternal education are determinants of rotavirus vaccine uptake | Vaccine-specific: Applies the multiple determinant model to a specific vaccine./Positivist decomposition assumes inequalities are factorially attributable, but overlooks intersectionality | The Enduring Triad: Hegemony of Quantitative Lens  Model's behavioral framing is Western-centric, potentially missing cultural determinants in Ethiopia. |
| 47 | Wondimu et al. (2021) | Nationwide | This work was supported by Netherlands Organization for International Cooperation in Higher Education, Netherlands (Grant Number: NFP - PhD.16/0015). | Quantitative (Secondary)Secondary | 1,438/Not mentioned | Health science | Public health/Social Determinants of Health | Equity language used | Antenatal care coverage use, institutional delivery, and media exposure | Antenatal care coverage use, institutional delivery, media exposure, and maternal educational determinants of measles vaccine uptake | Vaccine Analysis: Repeats the standard determinant model for a different vaccine. | The Enduring Triad: Hegemony of Quantitative Lens  Stagnant socioeconomic gradients in measles vaccine uptake despite national coverage gains in Ethiopia signal the need for equity-explicit, pro-poor immunization strategies to achieve elimination goals |
| 48 | Wuneh et al. (2022) | Amhara, Oromia, Tigray &SNNP | Bill & Melinda Gates Foundation (INV-009691) to the London School of Hygiene and Tropical Medicine. | Quantitative (Secondary) | 10785 | Health sciences | Social  determinants | Equity language used | Poverty, education, access, service quality | Maternal and child health service inequities are linked to poverty, lack of education, lack of access, and poor-quality services. | Descriptive without Structural Analysis: Identifies some service barriers but lacks a deeper analysis of political structures sustaining them. | Disciplinary Narrowness: Hegemony of Quantitative Lens |
| 49 | Wuneh et al. (2022) | Tigray | The study was funded by the Bill and Melinda Gates Foundation (INV-009691) through the London School of Hygiene and Tropical Medicine. | Qualitative (Focus group and interviews | 22 in-depth interviews & 3 focus group discussions./48 participants: 24 IDIs (12 mothers with recent births, 12 husbands/fathers) + 4 FGDs (6–8 participants each: 2 with mothers, 2 with Health Development Army leaders). | Health Sciences | Health services/WHO Social Determinants of Health (SDH) framework and Thaddeus & Maine’s Three Delays Model; | Poverty, education, access, quality | Maternal and child health service inequities are linked to poverty, lack of education, lack of access, and poor-quality services. | Maternal and child health service inequities are linked to poverty, lack of education, lack of access, and poor-quality services./Inequities are socially produced; education, transport subsidies, and community empowerment are critical for equity. | Qualitative Insights (although Limited): Identifies barriers descriptively but lacks deeper political economy analysis of their root causes./Interpretivist, participant-centered approach privileges lived experience and social context over statistical averages | ,Positive exemplar of Qualitative insights into social determinants of maternal and child health inequities in sub-Saharan Africa). |
| 50 | Wuneh et al. (2019) | 4 Regions | The source of funding was the Bill& Melinda Gates Foundation (OPP1132551) through the London School of Hygiene and Tropical Medicine and the Ethiopian Public Health Institute. | Quantitative (Secondary) | 6,321  A total of 6000 households were sampled, but data were collected from 5714 households. | Health sciences | Health services | Equity language used | Wealth, maternal and child health | Wealth is the determinant of maternal, neonatal, and child health services utilization. | Wealth Focus: Reiterates wealth as the primary determinant across multiple health services. | The Enduring Triad: Hegemony of Quantitative Lens |
| 51 | Yaya et al. (2021) | Nationwide | No funding was received for this work. | Quantitative (Secondary) | 64,506/22,432 | Health science and social sciences | Social determinants | Equity language used | Socio-economic status, residence, and education | Low socio-economic status, rural residence, and being uneducated had a high chance of adolescent pregnancy and more births. | Adolescent Health: Applies the standard determinant model to adolescent sexual and reproductive health./Enduring socioeconomic and geographic inequities in adolescent fertility in Ethiopia hinder reproductive health goals, | The Enduring Triad: Hegemony of Quantitative Lens/: Positivist, DHS-centric approach assumes self-reported births and asset-based wealth objectively reflect fertility inequit |
| 52 | Yesuf & Calderon-Margalit (2013) | Nationwide | We would like to thank Measure DHS Corporation, Calverton, Maryland, USA, for providing the datasets for this investigation. ToTo acknowledge IMPH Program at the Braun School of Public Health, Hebrew University, Jerusalem, Israel, for providing access to databases and computers during the analysis of the datasets used in the study. | Quantitative (Secondary) | 7,978/23,193 | Health sciences | Public health/Andersen Behavioral Model of Health Services Use | Equity language used | Wealth, education, residence, region | Wealth, educational status, residence, and regional disparities are determinants of antenatal care coverage service utilization. | Comprehensive Determinants: Includes multiple variables but without integration into a coherent structural analysis. | The Enduring Triad: Hegemony of Quantitative Lens/Positivist, survey-driven approach assumes self-reported ANC reflects equitable access, but ignores quality, content, or provider bias. Equity framed statistically, not as rights or structural violence. |
| 53 | Yesuf et al. (2014) | Nationwide | We ¡acknowledge the support provided by the Braun School of Public Health, Hebrew University, Jerusalem, Israel, and Pears Foundation, Jerusalem, Israel, for providing computers, access to international peer-reviewed journals, and internet during data analysis. We also thank the Department of Health Services Management, Jimma University, Jimma, Ethiopia, for providing internet and access to HINARI, the WHO database of peer-reviewed Journals and Books, during manuscript preparation. We extend our gratitude to ICF International and CSA Ethiopia for granting access to the datasets used in this study./No fund declared | Quantitative (Secondary) | 28,927 | Health science | Health services | Equity language used | Wealth, residence, education | Wealth, residence, and educational status are determinants of institutional delivery utilization. | Delivery Care Focus: Standard determinant analysis applied to delivery care./positivist, survey-centric equity measurement assumes self-reported delivery | The Enduring Triad: Hegemony of Quantitative Lens |
| 54 | Yitbarek et al. (2021) | Jimma, Southwest Ethiopia | Jimma  University | Quantitative (Primary) | 835 | Health science | Health services utilization | Equality measurement | age, place of residence, and educational  status, ethnicity, religion, and occupation | Wealth and maternal educational status are determinants of neonatal health service utilization. | Decontextualized Local Application: Replicates standard individual-level model without local political/cultural contextualization | Hegemony of Quantitative Lens, Enduring Triad |
